# Supplementary material for: Overweight and obesity in south central Uganda: A population-based study
Source: PLOS Glob Public Health. 2022 Nov 21;2(11):e0001051. doi: 10.1371/journal.pgph.0001051 (PMC10021145; doi:10.1371/journal.pgph.0001051)
Supplement: S1 File — Table A. Combined logistic regression model of overweight on covariates. Table B. Combined logistic regression model of obesity on covariates. Table C. Combined logistic regression model of underweight on covariates. (DOCX) [file pgph.0001051.s001.docx]

**S1 File**

**Table A: Combined models for overweight and sex coefficient**

| Covariates | Adjusted RR [95% conf. interval] | p-value |
| --- | --- | --- |
| Age Group, years |  |  |
| 15-19 (Ref.) | -- | -- |
| 20-29 | 1.89, [1.64, 2.18] | <0.001 |
| 30-39 | 2.70, [2.33, 3.13] | <0.001 |
| 40+ | 2.78, [2.39, 3.23] | <0.001 |
|  |  |  |
| Community Type |  |  |
| Agrarian (Ref.) | -- | -- |
| Fishing | 1.34, [1.25, 1.44] | <0.001 |
| Trading | 1.15, [1. 09, 1.22] | <0.001 |
|  |  |  |
| SES group |  |  |
| Lowest (Ref.) | -- | -- |
| Low-middle | 1.39, [1.29, 1.51] | <0.001 |
| High-middle | 1.68, [1.56, 1.81] | <0.001 |
| Highest | 1.80, [1.67, 1.95] | <0.001 |
|  |  |  |
| Occupation |  |  |
| Agriculture/housework (Ref.) | -- | -- |
| Bar/Restaurant | 1.33, [1.21, 1.47] | <0.001 |
| Fishing | 1.28, [1.07, 1.54] | <0.001 |
| Trade/Shopkeeper | 1.44, [1.35, 1.53] | <0.001 |
| Other | 1.12, [1.04, 1.20] | 0.002 |
|  |  |  |
| Marital Status |  |  |
| Never married (Ref.) | -- | -- |
| Previously married | 1.33, [1.19, 1.50] | <0.001 |
| Currently married | 1.49, [1.34, 1.65] | <0.001 |
|  |  |  |
| Gender |  |  |
| Female (Ref.) | -- | -- |
| Male | 0.24, [0.18, 0.34] | <0.001 |
|  |  |  |
| Education |  |  |
| None (Ref.) | -- | -- |
| Primary | 0.91, [0.82, 1.01] | 0.073 |
| Secondary/Tertiary | 1.01, [0.91, 1.13] | 0.821 |
|  |  |  |
| Gender Education Interaction |  |  |
| Male#None (Ref.) | -- | -- |
| Male#Primary | 1.24, [0.89, 1.73] | 0.201 |
| Male#Secondary/Tertiary | 1.39, [0.99, 1.96] | 0.054 |
|  |  |  |
| Smoking status |  |  |
| No (Ref.) | -- | -- |
| Yes | 0.81, [0.68, 0.96] | 0.015 |
|  |  |  |
| Gender-smoke interaction |  |  |
| Male#No (Ref.) | -- | -- |
| Male#Yes | 0.43, [0.31, 0.60] | <0.001 |
|  |  |  |
| Religion |  |  |
| Muslim (Ref.) | -- | -- |
| Non-Muslim | 0.87, [0.81, 0.93] | <0.001 |
|  |  |  |
| Drinks Alcohol |  |  |
| No (Ref.) | -- | -- |
| Yes | 1.16, [1.06, 1.27] | 0.001 |
|  |  |  |
| Last alcoholic drink |  |  |
| >1 year (Ref.) | -- | -- |
| <1 month | 0.96, [0.86, 1.06] | 0.408 |
| <1 week | 1.03, [0.94, 1.13] | 0.523 |

**Table A:** Combined logistic regression model of overweight on covariates. RR = risk ratio. CI = Confidence Interval. SES = socioeconomic status. HIV = Human Immunodeficiency Virus. Ref. = reference group. Final model was adjusted for all other variables, including the interaction terms listed.

**Table B: Combined models for obesity sex coefficient**

| Covariate | Adjusted Risk Ratio [95% conf. interval] | p-value |
| --- | --- | --- |
| Age Group, years |  |  |
| 15-19 (Ref.) | -- | -- |
| 20-29 | 3.29, [2.19, 4.93] | <0.001 |
| 30-39 | 6.61, [4.39, 9.96] | <0.001 |
| 40+ | 7.06, [4.67, 10.68] | <0.001 |
|  |  |  |
| Gender |  |  |
| Female (Ref.) | -- | -- |
| Male | 0.14, [0.08, 0.24] | <0.001 |
|  |  |  |
| Community Type |  |  |
| Agrarian (Ref.) | -- | -- |
| Fishing | 1.54, [1.30, 1.82] | <0.001 |
| Trading | 1.26, [1.11, 1.44] | <0.001 |
|  |  |  |
| Gender-Community type interaction |  |  |
| Male#Agrarian | -- | -- |
| Male#Fishing | 1.21, [0.67, 2.21] | 0.526 |
| Male#Trading | 0.96, [0.60, 1.56] | 0.883 |
|  |  |  |
| SES group |  |  |
| Lowest (Ref.) | -- | -- |
| Low-middle | 1.58, [1.31, 1.89] | <0.001 |
| High-middle | 2.26, [1.91, 2.68] | <0.001 |
| Highest | 2.48, [2.07, 2.96] | <0.001 |
|  |  |  |
| Occupation |  |  |
| Agriculture/housework (Ref.) | -- | -- |
| Bar/Restaurant | 1.71, [1.39, 2.10] | <0.001 |
| Fishing | 0.84, [0.39, 1.83] | 0.662 |
| Trade/Shopkeeper | 1.87, [1.64, 2.14] | <0.001 |
| Other | 1.20, [1.03, 1.40] | 0.021 |
|  |  |  |
| Marital Status |  |  |
| Never married (Ref.) | -- | -- |
| Previously married | 1.57, [1.20, 2.06] | 0.001 |
| Currently married | 1.65, [1.29, 2.12] | <0.001 |
|  |  |  |
| Religion |  |  |
| Muslim (Ref.) | -- | -- |
| Non-Muslim | 0.80, [0.68, 0.93] | 0.005 |
|  |  |  |
| Gender-Religion |  |  |
| Male#Muslim (Ref.) | -- | -- |
| Male#Christian/Non-Muslim | 0.79, [0.43, 1.42] | 0.424 |
|  |  |  |
| Smoking status |  |  |
| No (Ref.) | -- | -- |
| Yes | 0.81, [0.57, 1.15] | 0.237 |
|  |  |  |
| Gender-smoke interaction |  |  |
| Male#No (Ref.) | -- | -- |
| Male#Yes | 0.41, [0.16, 1.06] | 0.065 |
|  |  |  |
| HIV status |  |  |
| Negative (Ref.) | -- | -- |
| Positive | 0.59, [0.51, 0.69] | <0.001 |
|  |  |  |
| Gender-HIV interaction |  |  |
| Male#Negative (Ref.) | -- | -- |
| Male#Positive | 0.83, [0.39, 1.73] | 0.614 |
|  |  |  |
| Drinks Alcohol |  |  |
| No (Ref.) | -- | -- |
| Yes | 1.10, [0.88, 1.36] | 0.413 |
|  |  |  |
| Gender-Drinks Alcohol interaction |  |  |
| Male#No (Ref.) | -- | -- |
| Male#Yes | 1.33, [0.57, 3.12] | 0.513 |
|  |  |  |
| Last Alcoholic drink |  |  |
| >1 year (Ref.) | -- | -- |
| <1 month | 1.02, [0.79, 1.32] | 0.878 |
| <1 week | 1.34, [1.06, 1.68] | 0.013 |
|  |  |  |
| Gender-Last Alcoholic Drink interaction |  |  |
| Male#More one year (Ref.) | -- | -- |
| Male#less than 1 month | 0.67, [0.23, 1.93] | 0.453 |
| Male#less than one week | 0.68, [0.30, 1.54] | 0.356 |

**Table B:** Combined logistic regression model of obesity on covariates. RR = risk ratio. CI = Confidence Interval. SES = socioeconomic status. HIV = Human Immunodeficiency Virus. Ref. = reference group. Final model was adjusted for all other variables, including the interaction terms listed.

**Table C: Combined models for underweight sex coefficient**

| Covariates | Adjusted RR [95% CI] | p-value |
| --- | --- | --- |
| Age Group, years |  |  |
| 15-19 (Ref.) | -- | -- |
| 20-29 | 0.47, [0.41, 0.54] | <0.001 |
| 30-39 | 0.46, [0.38, 0.56] | <0.001 |
| 40+ | 0.65, [0.54, 0.79] | <0.001 |
|  |  |  |
| Community Type |  |  |
| Agrarian (Ref.) | -- | -- |
| Fishing | 0.57, [0.48, 0.68] | <0.001 |
| Trading | 0.92, [0.83, 1.02] | 0.116 |
|  |  |  |
| SES group |  |  |
| Lowest (Ref.) | -- | -- |
| Low-middle | 0.79, [0.71, 0.89] | <0.001 |
| High-middle | 0.62, [0.54, 0.71] | <0.001 |
| Highest | 0.62, [0.55, 0.70] | <0.001 |
|  |  |  |
| Occupation |  |  |
| Agriculture/housework (Ref.) | -- | -- |
| Bar/Restaurant | 0.93, [0.67, 1.30] | 0.673 |
| Fishing | 0.52, [0.39, 0.70] | <0.001 |
| Trade/Shopkeeper | 0.59, [0.49, 0.72] | <0.001 |
| Other | 1.02, [0.92, 1.14] | 0.660 |
|  |  |  |
| Marital Status |  |  |
| Never married (Ref.) | -- | -- |
| Previously married | 0.80, [0.66, 0.96] | 0.019 |
| Currently married | 0.68, [0.58, 0.80] | <0.001 |
|  |  |  |
| Gender |  |  |
| Female (Ref.) | -- | -- |
| Male | 1.75, [1.57, 1.97] | <0.001 |
|  |  |  |
| Smoking status |  |  |
| No (Ref.) |  |  |
| Yes | 1.51, [0.98, 2.33] | 0.060 |
|  |  |  |
| Gender-smoke interaction |  |  |
| Male#No (Ref.) | -- | -- |
| Male#Yes | 1.33, [0.84, 2.10] | 0.217 |
|  |  |  |
| HIV status |  |  |
| Negative (Ref.) | -- | -- |
| Positive | 1.55, [1.28, 1.88] | <0.001 |
|  |  |  |
| Gender-HIV interaction |  |  |
| Male#Negative (Ref.) | -- | -- |
| Male#Positive | 0.84, [0.65, 1.09] | 0.190 |
|  |  |  |
| Drinks Alcohol |  |  |
| No (Ref.) | -- | -- |
| Yes | 0.91, [0.75, 1.11] | 0.375 |
|  |  |  |
| Last Alcoholic drink |  |  |
| >1 year (Ref.) | -- | -- |
| <1 month | 0.99, [0.77, 1.27] | 0.926 |
| <1 week | 1.03, [0.84, 1.27] | 0.776 |

**Table C:** Combined logistic regression model of underweight on covariates. RR = risk ratio. CI = Confidence Interval. SES = socioeconomic status. HIV = Human Immunodeficiency Virus. Ref. = reference group. Final model was adjusted for all other variables, including the interaction terms listed.
