# Supplementary material for: Overweight and obesity in south central Uganda: A population-based study
Source: PLOS Glob Public Health. 2022 Nov 21;2(11):e0001051. doi: 10.1371/journal.pgph.0001051 (PMC10021145; doi:10.1371/journal.pgph.0001051)
Supplement: Attachment — Submitted filename: Response to Reviewers.docx [file pgph.0001051.s002.docx]

Point by point response: PLOS Global Public Health

**Overweight and Obesity in South Central Uganda: A Population-Based Study**

Journal requirements

1. A funding statement has been included in the online portal:
   a. Funding: This work was supported by the National Heart, Lung, and Blood Institute (R01HL107275), the Division of Intramural Research, National Institute of Allergy and Infectious Diseases, National Institutes of Health, the Swedish Research Council (grant numbers 2015-05864, 2016-05647), and the Johns Hopkins University Center for AIDS Research (P30AI094189). The funders had no role in study design, data collection and analysis, decision to publish, or preparation of the manuscript.
2. The references have been reviewed and no cited articles have been retracted.

Reviewer 1 comments

Comment 1. Title: I feel that the title is misleading since this is a cross-sectional study within a cohort study. Remove cohort from title:

- We feel that this comment is valid, as the study is using cross-sectional data from a longitudinal cohort study. Therefore, “cohort” has been removed from the title.

Comment 2. Abstract: from the sentence starting with: "For female participants...." the Prevalence Risk Ratio (PRR) is used. However in the tables 5-7 the risk ratio (RR) is used. I am not sure which one is correct but expect it to be the RR:

- We appreciate this astute observation and agree that consistency is needed. In this paper, we are reporting the prevalence risk ratio (PRR) rather than risk ratio (RR) because the data analyzed is cross-sectional not longitudinal. Prevalence risk is more accurate. We have updated tables 5-7 to show PRR rather than RR.

Comment 3. Sample: Although it has been published elsewhere please provide a brief summary for the reader:

- Thank you for this comment requesting further expansion on the study design. We agree that this brief summary will be helpful for readers. Therefore, the following has been added in lines 90-96: “Briefly, RCCS holds informational community mobilization events and periodic censuses in the Rakai district, during which all households from the study communities are approached by the study team for recruitment. Eligible cohort participants are identified and enrolled. RCCS collects key health data of adult residents (aged 15 to 49 years) who provide written informed consent, using periodic interviews to assess demographics, sexual and health-seeking behaviors, and uptake of HIV prevention and treatment services.”

Comment 4. Line 87-89: provide the make and model of the anthropometer and scales and indicate how they were standardized:

- Thank you for this comment. This has been clarified in line 94, “SECA scale, model 762 1019008”. Height was measured using a stadiometer (described in lines 100-101)

Comment 5. Line 119: "To evaluate factors associated with underweight, overweight and obesity, we used a modified Poisson model with a log link function to estimate prevalence risk ratios (PRR)." Was this the best method to use regarding the fact that the Poisson method is mainly associated with cohort studies not cross-sectional ones?

- We appreciate this question as it voices a topic the team thought about deeply prior to selecting the final statistical technique. After discussion with the group statistician and review of the literature on best analytical methods for this kind of study, the modified Poisson model was chosen as the best way to calculate prevalence risk ratios in cross-sectional studies while avoiding convergence problems that arise with log-binomial models (see citation 1 below, citation 20 in manuscript).

Comment 6. Tables: Please provide full titles for all tables so that they can stand alone.

- The titles have been updated as follows:
- Table 1. Participants’ Demographics and Characteristics.
- Table 2. Female population by BMI category over sociodemographic characteristics, including mean BMI.
- Table 3. Male population by BMI category over sociodemographic characteristics, including mean BMI.
- Table 4: Prevalence risk ratio of underweight, overweight, and obesity by participant sex.
- Table 5. Predictors of Overweight/Obesity from adjusted and unadjusted multivariable analysis models for male and female participants.
- Table 6. Predictors of Obesity from adjusted and unadjusted multivariable analysis models for male and female participants.
- Table 7. Predictors of Underweight from adjusted and unadjusted multivariable analysis models for male and female participants

Comment 7. It is not clear what < 12 months, < one month, < one week means. Rather write this out:

- These labels have been clarified to the following categories— “more than 12 months”, “within the last 12 months”, “1-4 weeks”, and “0 days – 1 week”.

Comment 8. It is recommended that tables 2 and 3 provide an additional column for BMI>=25 instead of only describing this in the text.

- We agree with this reviewer comment as such an extra column would supplement information already described in the text. An additional column of combined overweight/obese participants has been added to tables 2 and 3.

Comment 9. From Table 4 on please note the variables which have been adjusted for under each table.

- This has been addressed with the following addition beneath table 4: “Adjusted for age group, community type, socioeconomic status, occupation, marital status, religion, smoking, current alcohol use, and/or last alcohol use. See supplemental information for full analysis models, including inclusion of interaction terms” (lines 212-214).
- Tables 5-7 list the variables not included in the final analysis. We added the following to each table to show that all other variables were included in the final model: “*All other variables listed were adjusted for in the final model.”*
- Additionally, the supplemental tables in the supplemental information provide full analysis models, including all variables adjusted for and interaction terms included in each of the three regression models.

Reviewer #2: Comments to authors

Comment 1. In the methodology it was not clear which of the category of BMI was used as reference in the case of overweight/obesity and in the case of obesity as well as underweight. Because of this when the authors were describing the results the comparison did not quite come clearly.

- Thank you for this clarification comment. The multivariable regression analysis was done separately for each BMI category. Within each regression model, there was no reference BMI category. Instead, each variable had a reference group chosen and used as the comparison group. For example, when looking at the prevalence risk of overweight/obesity in females by the variable of age, we used the age group 15-19 years as the reference group. Therefore, by setting the risk of overweight/obesity in females age 15-19 as 1.0, we could interpret the prevalence risk ratios as the prevalence risk of overweight/obesity for a female of “Y age group” relative to the prevalence risk of overweight/obesity for females aged 15-19. This reference strategy was used for all variables and all BMI categories. The reference group is indicated clearly for each variable in tables 5-7.

Comment 2. I was thinking that because the dependent had three categories’ authors could have used multinomial logistic regression to estimate the risk of developing each condition.

- The reviewer makes a good point that the BMI variable had three outcomes: underweight, overweight/obese, or obese. However, our primary outcome “being overweight and/or obese” is binary, (participant is overweight/obese, or participant not overweight/obese). Secondary outcomes in the study were “being underweight or not” and “being obese or not”. While we considered using a multivariable logistic regression model, this technique would provide odds ratios rather than risk ratios. In this cross-sectional analysis of overweight/obesity, where the outcomes are not rare, prevalence risk ratios were preferred over odds ratios. Additionally, log-binomial regression models were explored but ultimately inappropriate because of convergence issues that emerge with the statistical software. The modified Poisson regression has been identified in prior literature as appropriate and statistically rigorous for this kind of analysis (see citation 1 below, citation 20 in manuscript).
- The manuscript has been updated in lines 123 and 125 to describe the study’s primary and secondary outcomes more accurately.

Comment 3. Authors also used living in fishing community and fishing as different covariate to estimate the risk of overweight/obesity. I find these two variables as potential confounders which could mask, the effect of the other covariates as the probability of being a fisherman is high when one lives in a fishing community. Authors may want to explain why these two variables were kept together in the model.

- In this study, we used the variance inflation factor (VIF) as a measure of multi-collinearity to ensure there was not an unacceptable level of correlation between any of the variables included in the final models. All coefficients had a VIF less than 5, the threshold often cited in literature and statistical learning as concerning for collinearity (see citation 2 below). Therefore, we are reassured against significant levels of collinearity, even between fishing community and fishing occupation.
- Overall, we felt that both fishing community and fishing occupation should be included as variables in the final models. While occupation was relatively linked to community type due to historical categorizations of the communities listed in the study, it did not consistently predict a person’s community of residence. Fishers lived outside of fishing communities and many people living in fishing communities had occupations outside of fishing (in fact, only 31.4% percent of those residing in fishing communities worked in fishing).

Comment 4. Also, authors were also silent about living in fishing community and fishing as a risk factor of high BMI in the discussion. Authors may want to provide some explanation on the association of these variables (living in fishing community and fishing). Perhaps it may have to do with how fishing is done in Uganda.

- The reviewer points out an important association seen in the study, the higher prevalence risk of obesity for those in fishing communities and fishing occupations. This is discussed in lines 364 – 370, where we highlight the increased prevalence risk of obesity amongst participants with occupations in fishing, bar/restaurant work, and trading/shopkeeping. It is not clear why obesity is associated with the fishing occupation. The fishing occupation encompasses a wide variety of individuals in the fishing industry, ranging from fishermen going out on the lake, to boat owners, to fish traders, with variable levels of associated physical activity. Fishing is a relatively lucrative occupation which may increase access to calorie-rich foods. This association is something we will look to explore further in future studies.

Citations

Naimi AI, Whitcomb BW. Estimating Risk Ratios and Risk Differences Using Regression. American Journal of Epidemiology [Internet]. 2020 Jun 1 [cited 2022 Feb 2];189(6):508–10. Available from: <https://academic.oup.com/aje/article/189/6/508/5812650>

Gareth, J., Daniela, W., Trevor, H., & Robert, T. (2013). *An introduction to statistical learning: with applications in R*. Pg. 101-102. Spinger.
